# Updates in SJS/TEN: collaboration, innovation, and community

**DOI:** 10.3389/fmed.2023.1213889

**Published:** 2023-10-11

**Authors:** Madeline E. Marks, Ramya Krishna Botta, Riichiro Abe, Thomas M. Beachkofsky, Isabelle Boothman, Bruce C. Carleton, Wen-Hung Chung, Ricardo R. Cibotti, Roni P. Dodiuk-Gad, Christian Grimstein, Akito Hasegawa, Jay H. Hoofnagle, Shuen-Iu Hung, Benjamin Kaffenberger, Daniela Kroshinsky, Rannakoe J. Lehloenya, Michelle Martin-Pozo, Robert G. Micheletti, Maja Mockenhaupt, Keisuke Nagao, Suman Pakala, Amy Palubinsky, Helena B. Pasieka, Jonathan Peter, Munir Pirmohamed, Melissa Reyes, Hajirah N. Saeed, Jeffery Shupp, Chonlaphat Sukasem, Jhih Yu Syu, Mayumi Ueta, Li Zhou, Wan-Chun Chang, Patrice Becker, Teresa Bellon, Kemberlee Bonnet, Gianpiero Cavalleri, James Chodosh, Anna K. Dewan, Arturo Dominguez, Xinzhong Dong, Elena Ezhkova, Esther Fuchs, Jennifer Goldman, Sonia Himed, Simon Mallal, Alina Markova, Kerry McCawley, Allison E. Norton, David Ostrov, Michael Phan, Arthur Sanford, David Schlundt, Daniel Schneider, Neil Shear, Kanade Shinkai, Eric Tkaczyk, Jason A. Trubiano, Simona Volpi, Charles S. Bouchard, Sherrie J. Divito, Elizabeth J. Phillips

**Affiliations:** ^1^Center for Drug Interactions and Immunology, Division of Infectious Disease, Department of Medicine, Vanderbilt University Medical Center, Nashville, TN, United States; ^2^Division of Dermatology, Niigata University Graduate School of Medical and Dental Sciences, Niigata, Japan; ^3^Departments of Dermatology and Medicine, Uniformed Services University, Bethesda, MD, United States; ^4^The SFI Centre for Research Training in Genomics Data Science, Dublin, Ireland; ^5^Division of Translational Therapeutics, Department of Pediatrics, Faculty of Medicine, University of British Columbia and the British Columbia Children’s Hospital Research Institute, Vancouver, BC, Canada; ^6^Department of Dermatology, Drug Hypersensitivity Clinical and Research Center, Chang Gung Memorial Hospital, Taoyuan, Taiwan; ^7^National Institute of Arthritis and Musculoskeletal and Skin (NIAMS), National Institutes of Health (NIH), Bethesda, MD, United States; ^8^Department of Dermatology, Emek Medical Center, Afula, Israel; ^9^Division of Dermatology, Department of Medicine, University of Toronto, Toronto, ON, Canada; ^10^Department of Dermatology, Bruce Rappaport Faculty of Medicine, Technion Institute of Technology, Haifa, Israel; ^11^Office of Clinical Pharmacology, Office of Translational Sciences, Center for Drug Evaluation and Research, U.S. Food and Drug Administration, Silver Spring, MD, United States; ^12^Liver Disease Research Branch, Division of Digestive Diseases and Nutrition of NIDDK, National Institutes of Health (NIH), Bethesda, MD, United States; ^13^Cancer Vaccine and Immune Cell Therapy Core Laboratory, Department of Medical Research, Chang Gung Memorial Hospital, Taoyuan, Taiwan; ^14^Department of Dermatology, Ohio State University Wexner Medical Center, Columbus, OH, United States; ^15^Department of Dermatology, Brigham and Women’s Hospital, Harvard Medical School, Boston, MA, United States; ^16^Division of Dermatology, Department of Medicine, University of Cape Town, Cape Town, South Africa; ^17^Department of Dermatology, Perelman School of Medicine, University of Pennsylvania, Philadelphia, PA, United States; ^18^Dokumentationszentrum schwerer Hautreaktionen (dZh), Department of Dermatology, Medical Center and Medical Faculty, University of Freiburg, Freiburg, Germany; ^19^The Burn Center, MedStar Washington Hospital Center, Washington, D.C., DC, United States; ^20^Department of Dermatology, MedStar Health/Georgetown University, Washington, D.C., DC, United States; ^21^Division of Allergy and Clinical Immunology, Department of Medicine, University of Cape Town, Cape Town, South Africa; ^22^Department of Pharmacology and Therapeutics, University of Liverpool, Liverpool, United Kingdom; ^23^Center for Drug Evaluation and Research, United States Food and Drug Administration, Silver Spring, MD, United States; ^24^Massachusetts Eye and Ear, Harvard Medical School, Boston, MA, United States; ^25^Department of Surgery, Plastic and Reconstructive Surgery, Biochemistry, and Molecular and Cellular Biology, MedStar Washington Hospital Center, Georgetown University School of Medicine, Washington, D.C., DC, United States; ^26^Department of Pathology, Faculty of Medicine Ramathibodi Hospital, Mahidol University, Bangkok, Thailand; ^27^Department of Cell Biology and Anatomy, College of Medicine, National Cheng Kung University, Tainan, Taiwan; ^28^Department of Frontier Medical Science and Technology for Ophthalmology, Kyoto Prefectural University of Medicine, Kyoto, Japan; ^29^Division of General Internal Medicine and Primary Care, Brigham and Women’s Hospital, Harvard Medical School, Boston, MA, United States; ^30^Division of Allergy, Immunology, and Transplantation, National Institute of Allergy and Infectious Disease, Bethesda, MD, United States; ^31^Drug Hypersensitivity Laboratory, La Paz Health Research Institute (IdiPAZ), Madrid, Spain; ^32^Department of Psychology, Vanderbilt University, Nashville, TN, United States; ^33^University of New Mexico School of Medicine, Albuquerque, NM, United States; ^34^Department of Dermatology, Vanderbilt University Medical Center, Nashville, TN, United States; ^35^Department of Dermatology and Internal Medicine, UT Southwestern Medical Center, Dallas, TX, United States; ^36^Department of Neuroscience, Johns Hopkins University School of Medicine, Baltimore, MD, United States; ^37^Department of Cell, Developmental, and Regenerative Biology and Dermatology, Black Family Stem Cell Institute, Mount Sinai School of Medicine, New York, NY, United States; ^38^Department of Obstetrics and Gynecology, University of Washington, Seattle, WA, United States; ^39^Division of Pediatric Infectious Diseases and Clinical Pharmacology, Children’s Mercy, Kansas City, MO, United States; ^40^College of Medicine, University of Cincinnati, Cincinnati, OH, United States; ^41^Division of Infectious Diseases, Department of Medicine, Vanderbilt University Medical Center, Nashville, TN, United States; ^42^Department of Dermatology, Memorial Sloan Kettering Cancer Center, Weill Cornell Medical College, New York, NY, United States; ^43^Stevens-Johnson Syndrome Foundation, Westminster, CO, United States; ^44^Division of Pediatric Allergy, Immunology, and Pulmonary Medicine, Department of Pediatrics, Vanderbilt University Medical Center, Nashville, TN, United States; ^45^Department of Pathology, Immunology and Laboratory Medicine, University of Florida, Gainesville, FL, United States; ^46^Division of Pharmacovigilance-I, Center for Drug Evaluation and Research, U.S. Food and Drug Administration, Silver Spring, MD, United States; ^47^Division of Trauma, Surgical Critical Care, and Burns, Loyola University Medical Center, Chicago, IL, United States; ^48^Department of Psychiatry and Surgery, MedStar Washington Hospital Center, Georgetown University School of Medicine, Washington, D.C., DC, United States; ^49^Department of Dermatology, University of California, San Francisco, San Francisco, CA, United States; ^50^Department of Veterans Affairs, Vanderbilt Dermatology Translational Research Clinic (VDTRC.org), Nashville, TN, United States; ^51^Department of Infectious Diseases and Medicine, Austin Health, University of Melbourne, Melbourne, VIC, Australia; ^52^National Human Genome Research Institute (NHGRI), National Institutes of Health (NIH), Bethesda, MD, United States; ^53^Department of Opthalmology, Loyola University Medical Center, Chicago, IL, United States

**Keywords:** Stevens-Johnson Syndrome, Toxic Epidermal Necrolysis, severe adverse cutaneous drug reactions, HLA genotyping, pharmacogenomics, body surface area, electronic medical record, SCORTEN

## Abstract

Stevens-Johnson Syndrome/Toxic Epidermal Necrolysis (SJS/TEN) is a predominantly drug-induced disease, with a mortality rate of 15–20%, that engages the expertise of multiple disciplines: dermatology, allergy, immunology, clinical pharmacology, burn surgery, ophthalmology, urogynecology, and psychiatry. SJS/TEN has an incidence of 1–5/million persons per year in the United States, with even higher rates globally. One of the challenges of SJS/TEN has been developing the research infrastructure and coordination to answer questions capable of transforming clinical care and leading to improved patient outcomes. SJS/TEN 2021, the third research meeting of its kind, was held as a virtual meeting on August 28–29, 2021. The meeting brought together 428 international scientists, in addition to a community of 140 SJS/TEN survivors and family members. The goal of the meeting was to brainstorm strategies to support the continued growth of an international SJS/TEN research network, bridging science and the community. The community workshop section of the meeting focused on eight primary themes: mental health, eye care, SJS/TEN in children, non-drug induced SJS/TEN, long-term health complications, new advances in mechanisms and basic science, managing long-term scarring, considerations for skin of color, and COVID-19 vaccines. The meeting featured several important updates and identified areas of unmet research and clinical need that will be highlighted in this white paper.

## 1. Introduction

Stevens-Johnson Syndrome (SJS) and Toxic Epidermal Necrolysis (TEN) are life-threatening, immunologically-mediated, severe, cutaneous adverse drug reactions (IM-ADRs) (1). They are thought to be clinically and mechanistically one illness defined across a spectrum of severity and classified according to the extent of body surface area (BSA) detached: SJS (<10% BSA detached), SJS/TEN (10–30% BSA detached), and TEN (>30% BSA detached) (2). SJS/TEN has an overall mortality of 15–20% but can be more than 50% in the elderly and immunocompromised (2). The incidence rate for SJS/TEN is 1–5 cases per million persons annually in the developed world (3). These rates are likely even higher in the developing world, where many infectious diseases are endemic, and corresponding treatments include drugs that are commonly associated with SJS/TEN. Although SJS/TEN can have an underlying infectious etiology, it is more commonly related to small-molecule drug therapies in more than 80% of adults (4). Drug therapies with the highest risks include aromatic antiepileptic drugs, sulfonamide antibiotics, and allopurinol (1). A causality assessment tool, known as the algorithm of drug causality for EN (ALDEN), defines drugs with a score of 4 or higher as being at higher risk of being associated with SJS/TEN (5). Over the last two decades, research has revealed that drug-induced SJS/TEN is an HLA class I-restricted CD8+ T-cell mediated disease (6). Yet, most drugs still lack known HLA risk alleles and other genetic associations. For some drugs, an HLA risk allele defined in one population will not actually be the main HLA risk association generalizable across all populations. If a known risk HLA allele is present, however, the risk of developing SJS/TEN is thought to be equal across different races and ethnicities. More research is needed to gain a more comprehensive understanding of the genetic risk factors associated with SJS/TEN. Stereotyping and race-based testing for HLA risk is discouraged (6, 7).

**Figure 1:**
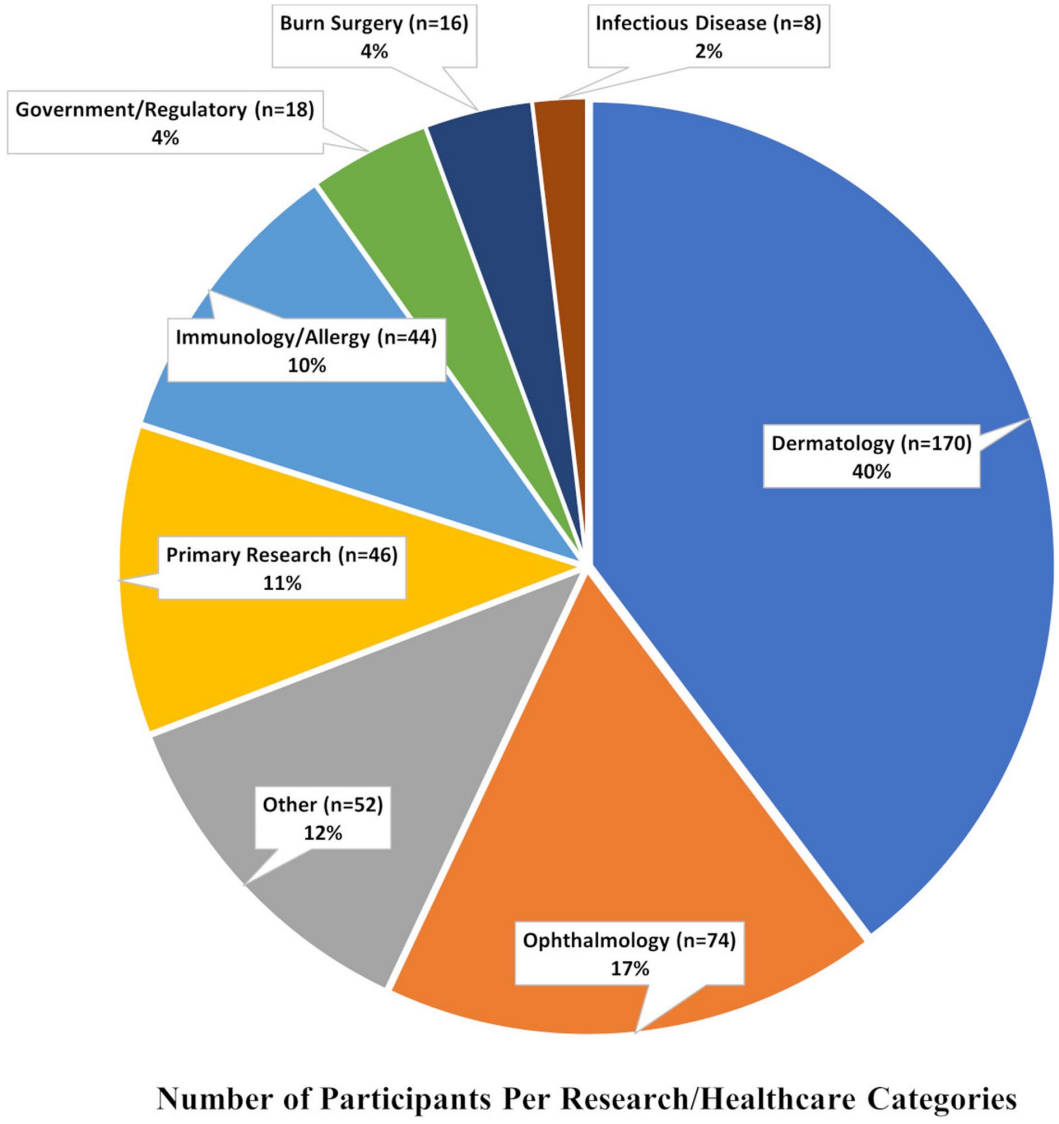
Pie chart representing the percentage of participants per research/healthcare categories.

Several conferences have furthered goals of increased mentoring and networking in the field of SJS/TEN. In 2021, a two-day virtual meeting titled “SJS/TEN 2021: Collaboration, Innovation, and Community” brought together scientists and community members (Figure 1) to promote awareness, review recent progress, and set priorities for improving patient outcomes (4, 6, 8). At this meeting, we were saddened to acknowledge the loss of a great leader in SJS/TEN: Professor Jean-Claude Roujeau (9) (Supplementary Figure). This international meeting was built on the success of previous conferences in 2017 (8) and 2019 (4) highlighting the cutting-edge research on the prediction, prevention, early diagnosis, and treatment of SJS/TEN. In this paper we review the current state of knowledge in the field, along with the future priorities for patients, providers, and researchers.

Improving outcomes and raising awareness for SJS/TEN requires community engagement and is extremely important for moving the field forward. Awareness among physicians and broad healthcare constituencies is essential to facilitating early identification, diagnosis, and accurate documentation of high-risk medications in the electronic health records (EHR) for SJS/TEN. Patients perceive that most providers are not appropriately trained in the recognition, early diagnosis, triage, or treatment of SJS/TEN. Part of the challenge is the lack of high-level evidence to support specific therapeutic interventions. However, across critical care, the implementation of supportive care has made the most difference in patient outcomes, which stands true today (10). Additionally, the development and distribution of standardized care plans for SJS/TEN would also be beneficial for mending this gap. Delphi-based consensus exercises have both supported a consensus on the best supportive care practice (11) for SJS/TEN. A survey of SJS survivors attending SJS/TEN 2021 identified several barriers to receiving the post-discharge information and care they need (12).

SJS/TEN patients have also stressed the need for a standardized care protocol for improving patient outcomes (Table 1). SJS/TEN patients and survivors are concerned with the provision of standardized guidelines, a multidisciplinary team, and universal protocols for eye care during the acute stage of SJS/TEN. Patients would benefit from a standardized evidence-based protocol for early transfer to specialized facilities, that include both dermatologic and intensive care, for diagnosis and treatment (6). Additionally, the development of take-home care guidelines, and the distribution of educational materials to medical teams, patients, and caregivers would help improve post-discharge outcomes (12).

**Table 1:** SJS survivorship and patient perspectives.

Themes	Community perspective	Physician perspective
Mental health	-Follow up care -Bridge between hospital care and follow-up care -Increase healthcare provider education for SJS/TEN PTSD -Address mental health and changes immediately after SJS/TEN -Assist through the recovery process -Implement mandatory mental wellness checks before discharge from the hospital and beyond -Address survivor’s guilt -Improve mental health/grief counseling for loved ones who lost an SJS/TEN patients -Provide grief counseling for your “lost life” and changed life -Discuss financial burden -Address low self-esteem	-Understand the psychological impact, and related long term health complications -Conduct qualitative and quantitative research to implicate in clinical care -Understand how the disease condition affects the individual (psychologically, interpersonally, vocationally, and overall quality of life) -Provide realistic expectations about challenges during hospitalization and after discharge -Provide a multidisciplinary support team (social work, psychiatry, psychology) -Provide proper discharge document with a list of medications -Ensure post-discharge follow-ups and counseling with survivors
Long-term health complications	-Improve education for healthcare professionals on residual side effects -Recognize SJS/TEN side effects -Improve treatment for all side effects (more than only eye care, esophageal care, skin care, live care, reproductive care, oral care, dental care) -Increase access to healthcare professionals who specialize with SJS/TEN patients (both in-person and telehealth appointments) -Increase/improve physician response time -Ease transfer of patient records -Develop and utilize an SJS/TEN identification checklist -Implement the use of educational materials by doctors (flyers, brochures, posters)	-Understand the various long-term health-related complications and their effects -Understand complications vary based on the severity of cases -Recognize that treatment options will change according to the case presentation -Increase collaborative research projects to study cases post SJS/TEN -Prioritize long-term follow-up of cases -Provide advice on referral centers -Standardize health checkups to identify complications -Increase collaborative and coordinative work among clinicians -Provide proper documentation for future referrals
Eye care	-Treatment during the acute stage -Treatment post SJS/TEN -Prompt treatment and diagnosis -Education on eye care treatment -Contact an eye care specialist -Aftercare and follow-up appointments	-Understanding treatment during acute stage is critical -Provide proper examination and care by specialists -Recognize treatment options should not be limited to topical steroids. Surgical procedures need to be considered when appropriate -Plan on decreasing the risk of infection and vision loss -Increase knowledge of advanced surgical and sutureless procedures
Long-term scarring	-Awareness of how scarring impacts SJS/TEN survivors (skin, eyes, organs) -How scarring changes over time (thickening) -Improved education for healthcare professionals -Eliminate the use of “Rare” to classify SJS/TEN -Educate patients post SJS/TEN about scarring -Prioritize early diagnosis -Provide second opinions from healthcare providers who have treated SJS/TEN -Implement mandatory certification on SJS/TEN and retraining -Provide examples of SJS/TEN scaring (at all stages from early identification)	-Research best practices to identify, early diagnose and treat SJS/TEN -Implement standard treatment protocols -Confirm diagnosis through histology -Determine specific signs that occur in the presence of certain medications -Have evidence-based studies to determine the casual drugs and treatment options
Children with no identifiable drug cause	-Bring awareness that over-the-counter products are medications -Create awareness about infections causing SJS/TEN and avoid accusing medications used to treat the first symptoms of SJS/TEN -Provide for mental health concerns -Look at genetic factors (HLA-b1502) -Create screenings	-Awareness and documentation of the causal factors -Knowledge of the possibility of life-threatening GI tract involvement when treating cases of SJS/TEN -Consider the usage of steroids and enteric feeding
Special considerations in skin of color	-Identify SJS/TEN in the acute stage -Acknowledge the difference between the appearance of SJS/TEN in the skin of color -Awareness of hyperpigmentation -Lack of visible blisters at the acute stage -Consider low visibility (lack of redness) of SJS/TEN presentation -Improve time to diagnosis -Improve education for healthcare providers of SJS/TEN in the skin of color -Implement a specific checklist for skin of color (purple-looking skin vs. red-looking skin) for identification	-Educate on dyspigmentation, skin changes, and different types of scarring -Understand disease effect on all types of skin cells -Change of practice: start counseling at the bedside -Improve interactions with patients, survivors, and families -Improve pharmacist education on common drug allergies -Improve response to queries or concerns of survivors -Provide detailed discharge instructions with frequent concerns (what products to use on skin, etc.)
Scientific advances in SJS/TEN	-Genetic testing -More research studies and increased patient/survivor participation -Gaining the patient perspective -Spread knowledge/awareness of new SJS/TEN treatments -Get more funding for SJS/TEN research -Bring more awareness of SJS/TEN -Eliminate the use of the word RARE -Increase box warnings -Increase funding to assist patients with SJS/TEN who are not financially stable	-Strengthen experimental models -Predict possible risks and validate signals -Capture cases, specimens, interoperable repositories -Promote consistency and quality in research methods -Use pharmacogenomics for drug safety -Integrate distributed databases/biobanks could enable biomarker discovery/validation, test monitoring/utility -Implement multicenter investigations to further understand management and treatment
Safety of COVID-19 vaccines	-Ensure that patients/survivors understand that COVID-19 vaccines are safe, including risks of COVID-19 vs. risk of vaccine -Develop education on potential complications of COVID-19 as an SJS/TEN survivor	-Answer vaccine-related queries-Educate on different responses to the vaccine -Ensure patients it is safe to get the COVID-19 vaccine -Address the misconceptions, hesitancy, and fear of getting the vaccine

Decreasing the time to diagnosis and immediate cessation of the most likely implicated drug(s) is critical (6). Additionally, documenting all potentially implicated drugs in the EHR is imperative to ensure future drug safety. Optimization of specialized protocols, such as eye care, is necessary to reduce long-term ocular complications like blindness. Early engagement of a multidisciplinary team comprised of dermatology, ophthalmology, gynecology, urology, pulmonology, gastroenterology, psychology and/or psychiatry, and pharmacy is also essential to the creation of an effective rehabilitation plan. Such a plan should be decided directly upon admission to preserve a patient’s quality of life.

Another key issue for SJS/TEN is the lack of appropriate follow-up post-discharge. Patients need guidance on proper follow-up care from knowledgeable professionals to ensure physical, mental, and emotional recovery. Follow-ups with specialists and discharge materials, like a list of low versus high-risk drugs, are vital. Another priority voiced by SJS/TEN survivors and their families were referrals, by providers, to community and psychosocial support groups. These groups, whether face-to-face or online, would help to facilitate continued engagement and education following discharge from acute care (12).

## 2. Preventive efforts

### 2.1. Advances in SJS/TEN pharmacogenomics

**Figure 2:**
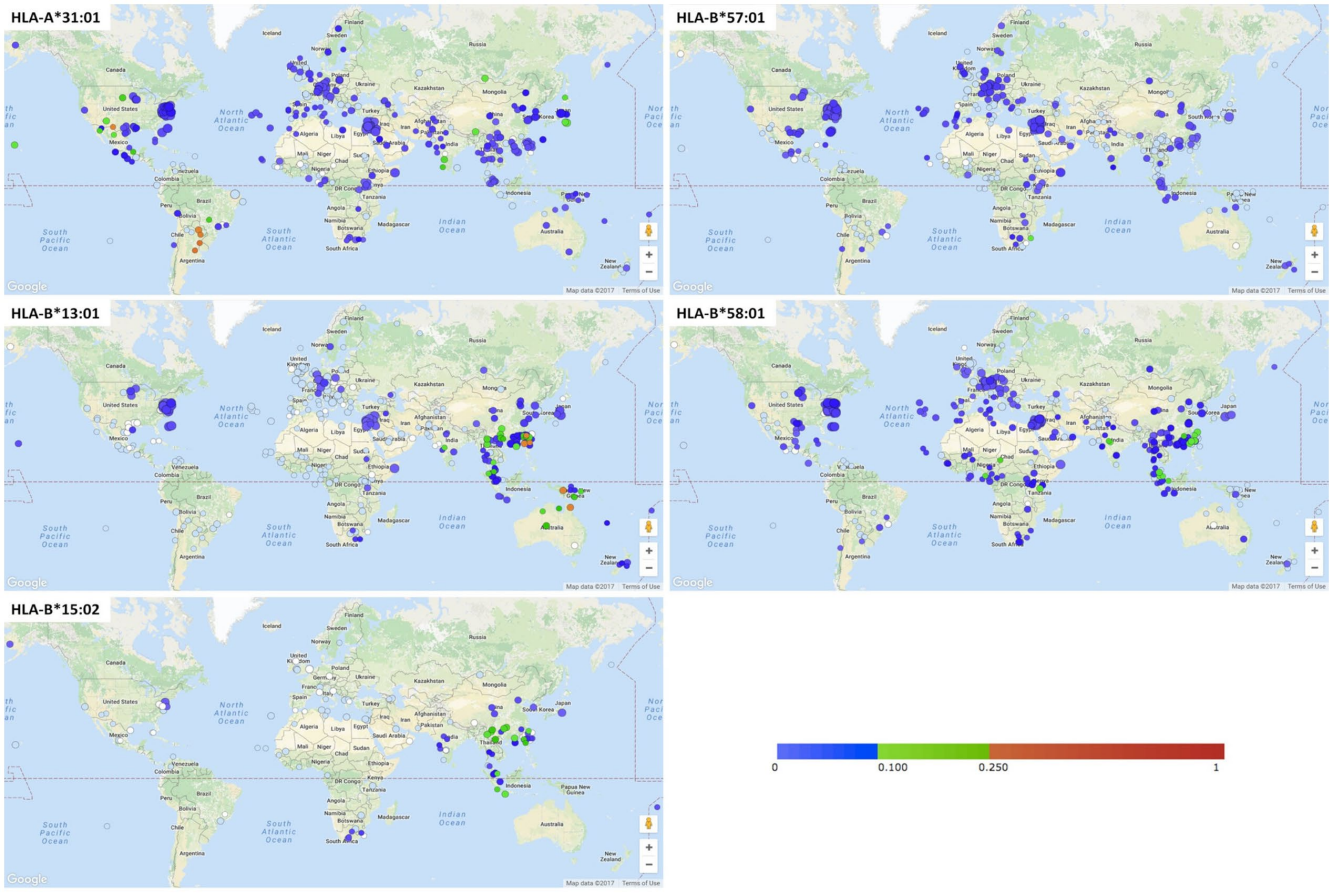
HLA risk alleles associated with SCAR in different ethnic populations.

Clinical implementation and assessment for pharmacogenetic risk markers before initiating drugs suspected of causing severe cutaneous adverse reactions (SCARs) has added significantly to prevention and diagnosis. Several medical centers worldwide have implemented clinical pharmacogenetic services with an aim to prevent SCARs, including SJS/TEN, and have reported on this experience (13–18). The preliminary results of large-scale prospective pharmacogenetic screenings conducted in Southeast Asia have substantially reduced rates of SCARs (19). HLA-B*15:02 genotyping prior to carbamazepine administration was found to be a cost-effective means to preventing carbamazepine-induced SJS/TEN. This has been shown in several, but not all, Asian countries (20), like Southeast and South Asian countries where the population has a higher HLA-B*15:02 allele frequency (5–20%), and a strong association between HLA-B*15:02 and SJS/TEN (21). The cost of HLA-B*15:02 screening is paid by national health insurance (Figure 2) in Hong Kong, Taiwan, Singapore (Chinese and Malay ethnicity), Thailand, and China (20). Caveats have been raised to the fact that the B75 serotype of HLA (which includes not only HLA-B*15:02 but HLA-B*15:21, HLA-B*15:08, HLA-B*15:11, HLA-B*15:30 and HLA-B*15:3) has been associated with carbamazepine SJS/TEN, however, the cost-effective single allele assays have been largely set-up to detect only HLA-B*15:02. Reports of carbamazepine SJS/TEN in patients carrying these other B75 HLA serotypes have been a primary reason in Southeast Asian countries for HLA-B*15:02 not detecting all patients at risk of developing carbamazepine SJS/TEN (22–25). Not all HLA alleles are associated with multiple clinical phenotypes of SCAR. For instance, HLA-B*58:01 is associated with both allopurinol SJS/TEN and drug reaction with eosinophilia and systemic symptoms/drug-induced hypersensitivity syndrome (DRESS/DIHS), however, HLA-B*15:02 is only associated with carbamazepine SJS/TEN. Therefore, even in Southeast Asia if an individual was negative for HLA-B*15:02 and other B75 HLA serotypes, they would still be at risk for carbamazepine DRESS/DIHS (Table 2) (26, 27) which has been associated with HLA-A*31:01.

**Table 2:** HLA class I risk alleles are shared amongst some but not all drugs & phenotypes.

Drug	HLA risk allele	MDE	DRESS/DIHS	SJS/TEN	DILI	HSS
Allopurinol	HLA-B*58:01					
Carbamazepine	HLA-B*15:02/B75 serotype					
Carbamazepine	HLA-A*31:01					
Dapsone	HLA-B*13:01					
TMP-SMX/Sulfapyridine	HLA-B*13:01					
Vancomycin	HLA-A*32:01					
Abacavir	HLA-B*57:01					
Flucloxacillin	HLA-B*57:01 HLA-B*57:03					

MDE, maculopapular drug eruption; DRESS/DIHS, drug reaction with eosinophilia and systemic symptoms/drug-induced hypersensitivity syndrome; SJS/TEN, stevens-johnson syndrome/toxic epidermal necrolysis; DILI, drug-induced liver injury; HSS, hypersensitivity syndrome.

**Figure 3:**
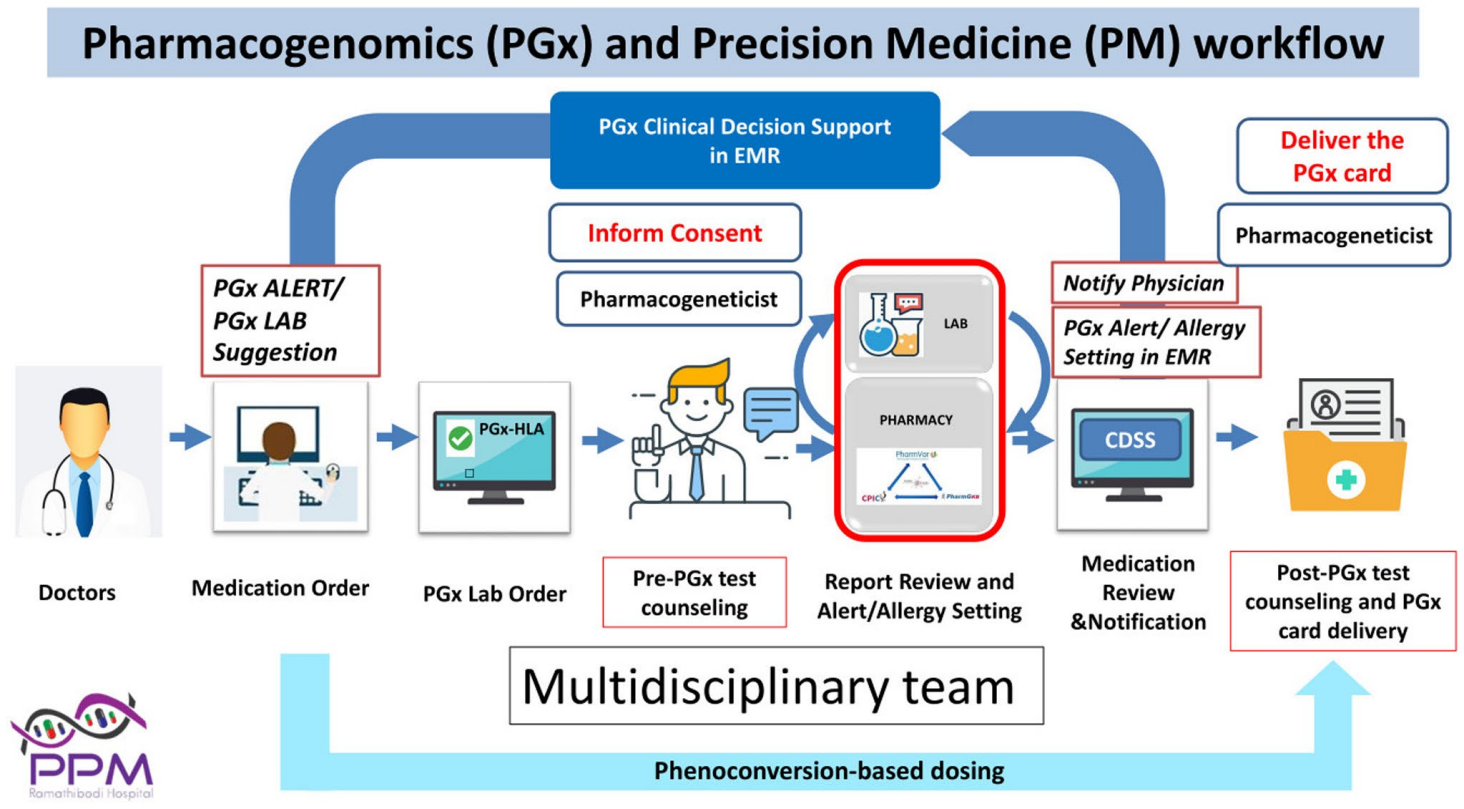
Pharmacogenomics test clinical workflow to alert physicians for drug prescriptions.

A model for precision medicine for the prediction and prevention of severe cutaneous adverse drug reactions (SCARs) including SJS/TEN has been the integration of pharmacogenetics into electronic health records (EHR) in Southeast Asian countries such as Thailand and Taiwan. The EHR-linked clinical decision support system (CDSS) improves the value of evidence-based pharmacogenetic screening through automated pop-up alerts that warn the prescriber if a high-risk allele is present (Figure 3). Diagnostic considerations and optimal treatment strategies are further offered so that clinicians are guided to choose lower-risk medications based on a patient’s genetic profile, without being overwhelmed by large amounts of clinical and genetic information (28). This approach has significantly reduced the incidence of specific drug-induced SJS/TEN in Taiwan and Thailand (20, 28).

The training curriculum for certification of proficiency in pharmacogenetics and precision medicine has gradually received greater attention and is now being incorporated into many medical schools and relevant postgraduate training programs. This curriculum has helped healthcare providers and trainees understand the importance of the clinical implementation of pharmacogenetics for the prediction and prevention of SJS/TEN (29). The pharmacogenetics course contains fundamental principles to provide knowledge on pharmacology (e.g., drug metabolism and pharmacokinetics) and human genetics/genomics (e.g., pathogenesis and polymorphism analysis). A practical approach is taken whereupon clinical decision-making strategies are built upon robust scientific evidence, clinical practice guidelines, and recommendations. Learning through case studies helps prescribers to become familiar with pharmacogenetic test interpretation and have confidence in incorporating the results into each patient’s healthcare management plan (30).

There are a growing number of clinical recommendations for pharmacogenetic tests used in clinical practice (31). Compared with a single test for a particular variant, the utilization of multiple-variant panels are considered beneficial since multiple risk variants can be screened for simultaneously. A pharmacogenetic panel containing multiple genetic variants that are significantly associated with an increased risk for developing SJS/TEN, or other SCAR, has been proposed and separately developed by research groups in Taiwan, Thailand, the UK, and Canada (19, 20, 30, 32). In a prospective observational study conducted in Southeast Asians (e.g., Taiwanese, Chinese, Thai, and Malaysian), the sensitivity and specificity of a multiple-variant panel for specific antiepileptic drugs (e.g., carbamazepine, oxcarbazepine, and phenytoin) was 75 and 90%, respectively (20). Although the less than 100% negative predictive value (NPV) means this would not be the perfect screening test, the results from the panel contribute to drug causality assessment. The panel is also helpful for identifying drugs with increased risk of SCARs to which the patient has not yet been exposed and making shared medical and therapeutic decisions with the patient. Therefore, the development of such multiple-variant pharmacogenetic panels is a dynamic and ongoing process, allowing for cost-efficient additions of newly discovered variants as the evidence base grows.

Given the low incidence of SJS/TEN, several international collaborations are underway to increase statistical power for identifying genetic variants and novel, but clinically relevant, pharmacogenetic associations across diverse ancestries. The latest scientific methods and technologies (e.g., GWAS meta-analysis, polygenic risk scoring, low-pass whole-genome sequencing) have the potential to make significant contributions to the field by uncovering increased genetic information, particularly for rare variants. More reliable evidence generated from real-world data, especially for under-served populations like First Nations, LatinX, and other diverse populations globally, remains an urgent need to advance the science of SJS/TEN research with regards to all ancestries.

To improve public health and drug safety, regulators update drug labeling and mandate boxed warnings to guide prescribers on the use of SJS/TEN suspect drugs. The U.S. Food and Drug Administration (FDA) has been proactive in incorporating pharmacogenetic risk factors in labeling. As of December 2020, 453 drug-biomarker pairs, including 311 drugs and 133 biomarkers, have been documented by the FDA, while 252 pairs are considered clinically actionable in SCAR. In the past, the recommendation for pharmacogenetic testing has varied based on the likelihood that SCAR, related to a specific drug, will occur in a specific population, and is largely based on the frequency of the HLA risk allele. As highlighted above to avoid structural racism and pharmacogenetic screening approaches that would disadvantage specific populations, a targeted approach based on provider stereotyped patient race is inaccurate. In addition, there has been widespread population admixture and the implications of a specific risk allele when present is the same regardless of the population (6). Other regulatory actions that have been taken by the Taiwanese FDA include collaboration with advisory committees, drug reporting centers that collect necessary safety data, and consultant experts who provide suggestions. A search for drugs which have a warning for SJS/TEN in the label can be done using the FDA label tool^1^.

## 3. Updates in diagnosis, assessment, and causality

### 3.1. General principles

The mainstay of SJS/TEN management is early clinical diagnosis and triage into a critical care setting with a high standard of supportive care, as discussed above. Histopathology aids in the clinical diagnosis and direct immunofluorescence helps identify autoimmune bullous disorders which can be confused with SJS/TEN particularly early in disease. All new drugs, and particularly those initiated within 4 days to 6 weeks, are suspect and should be discontinued (33). Early recognition is key. Although biological markers, such as granulysin, appear quite sensitive and specific for early identification of SJS/TEN, they lack widespread validation (34–36). An HLA risk allele, in addition to being a pre-prescription strategy that prevents SJS/TEN to specific drugs, may also add to the causality assessment that a specific drug is the culprit. Skin and patch testing generally have low sensitivity but high specificity for SJS/TEN with the exception of aromatic anticonvulsants which have a sensitivity of >50%. However, there is a range of sensitivity across different drugs from 0% (allopurinol) to >50% (aromatic anticonvulsants) (37, 38). *Ex vivo* and *in vitro* testing has had lower sensitivity than other severe cutaneous adverse drug reactions and needs more widespread validation and optimization (34, 39, 40). Rechallenge is contraindicated for all suspected culprit drugs and potentially cross-reactive drugs. The exception to this is the treatment of tuberculosis in low and middle-income countries where progress has been made using combinations of *ex vivo* testing and sequential additive challenges with methylprednisolone rescue (41, 42). Integrated approaches combining HLA typing, *in vivo* and *ex vivo*/*in vitro* testing have been advocated as having higher positive and negative predictive values than any one test alone (27, 42, 43).

### 3.2. Photography and artificial intelligence to improve SJS/TEN assessment

The SJS/TEN-specific severity-of-illness score (SCORTEN) has been the mainstay of measurements to define mortality risk of SJS/TEN in both clinical practice and research (44). The ABCD-10 (age, bicarbonate, cancer, dialysis, 10% BSA) is another cross-sectional severity scoring system that incorporated end-stage renal disease and was shown to perform slightly inferior to SCORTEN by underestimating mortality (45, 46). Another study proposed adding inflammatory markers to the SCORTEN to improve predictive accuracy. The only marker that was shown to improve predictive accuracy was the red cell width over hemoglobin ratio (47). More recently the CRISTEN (clinical risk score for TEN) was developed as a clinical risk score that does not require laboratory values and this initial study was validated across 416 patients multinationally (48). However, it must be realized that all of these scoring systems are cross-sectional tools weighed toward patient co-morbidities that measure severity at one point in time and are not useful for longitudinal assessments that measure changes in disease severity over time or the specific course of the disease. Due to the difficulties of undertaking randomized controlled trials in an uncommon and unpredictable disease, studies typically draw their primary outcome from a comparison of survival on therapy to the SCORTEN-predicted survival – the standardized mortality ratio for the therapy (49). Six of the seven SCORTEN prognostic factors are completely objective, drawing from irrefutable patient demography or quantitative physiologic or laboratory measurements. Coupled with these is a single subjective measure known as body surface area (BSA) of epidermal detachment, which was found to have a remarkable mortality association upon crossing a threshold of 10% BSA on the first day of hospitalization.

All clinical methods to estimate BSA have been shown to suffer major errors and inter-observer variations. For example, dermatology providers applying the rule of 9 s overestimated psoriatic plaque area by more than a factor of two in 49/80 patient assessments (50). Similarly, a meta-analysis of 26 studies in the burn literature found an average BSA estimation error of 70% across nearly 3,000 patients and concluded that neither the rule of 9 s nor palmar surface area are reliable estimates (51). Errors were significantly greater when under 20% BSA was affected. Notably, the rule of 9 s and more accurate Lund-Browder charts are both derived from paper-mâché molds from only 12 individuals (52). Very recently, our understanding of the human skin surface has been substantially advanced by high-resolution surface anthropometry laser body scans of 3,047 adults in the Civilian American and European Surface Anthropometry Resource (53), which proved that there is an enormous variability between individuals as to how much each body region contributes to the total BSA. Thus, regardless of evaluation by a dermatologist or in the burn unit, knowing the true BSA of an individual SJS/TEN patient is challenging. This represents a major barrier to the successful application of decades of clinical experience in SJS/TEN.

**Figure 4:**
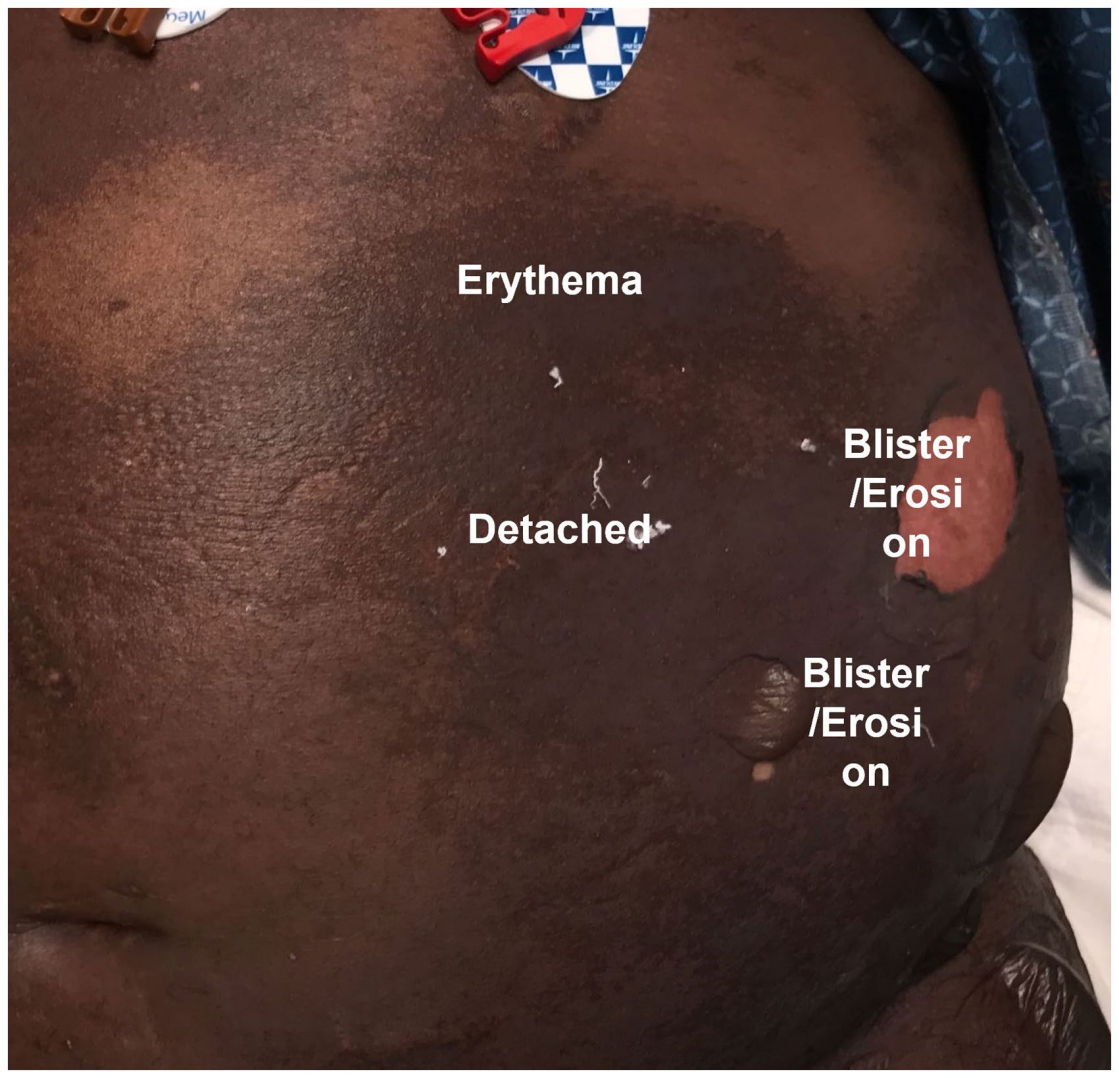
Example photograph of Vanderbilt Drug Safety patient (with permission) to guide standardized SJS/TEN scoring by illustrating the categorization of different appearances of skin into different terminology. Photo by Madeline Marks and Austin Cronin, VDTRC.org.

**Figure 5:**
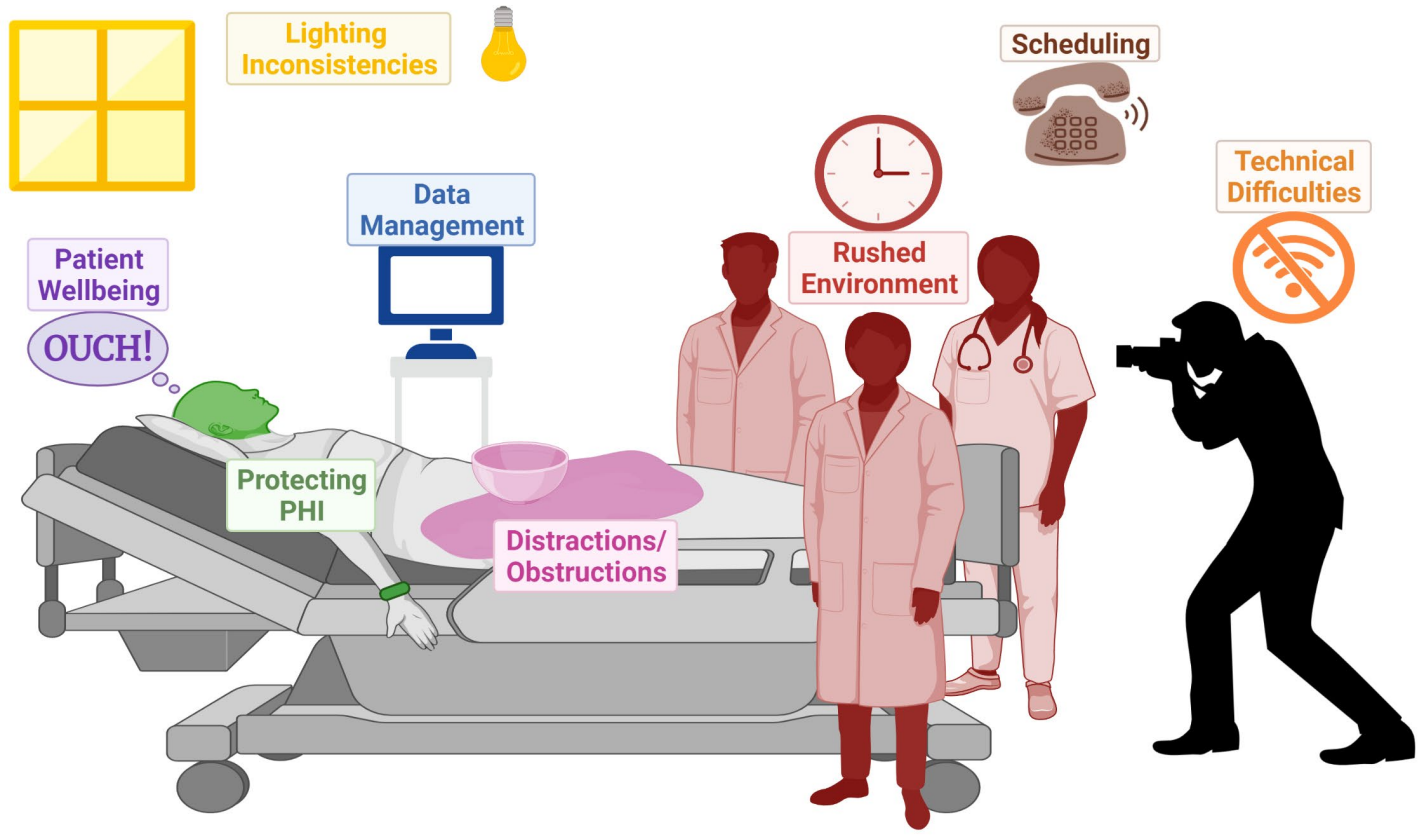
Infographic to illustrate the challenges of photographing in burn ICU.

Collection and analysis of SJS/TEN patient photos could serve an important role in addressing the gap presented by clinical BSA estimation variation. The development of standardized SJS/TEN-specific scoresheets with accompanying training and photos, including preferred terminology for different skin appearances (e.g., Figure 4), could be a major step forward in comparing the outcomes of individual patients and the results of different studies. For example, clinicians vary widely in whether they perform a Nikolsky sign or refer to dusky areas of erythema as detached skin. Photography-based adjudication that follows patient bedside BSA assessments, whether by the rater or another trained adjudicator, could further improve data quality. However, standardizing critically ill patient photography presents several challenges illustrated in Figure 5 and Table 3 and so may not be practical for all research groups. In this case, we recommend that future publications of SJS/TEN studies specify the primary data collection sheet used as well as detailed methods on how BSA was estimated. For example, the Lund-Browder method is more reliable than the rule of 9 s but may take more time (54). Ideally, the study would retain marked avatars and note the corresponding rater’s (or raters’) experience and specific training in BSA estimation.

**Table 3:** Challenges in photographing SJS/TEN patients.

Category	Challenge	Explanation	Solution
Room conditions	**Lighting inconsistency:** Variation in light tone and/or intensity, time of day, or weather.	Lighting inconsistencies increase the chance of shadowing, glare, and distorted skin tone in images.	Document the light sources in the room during the photo session. Consistently utilize the same device between sessions. Capture both flash and non-flash photos. Use portable light devices.
	**Rushed environment:** A high-stress intensive care environment caused by time constraints, simultaneous performance of procedures, photographer inexperience, or patient discomfort.	A rushed environment negatively impacts attention to detail and photography session quality.	Establish a relationship with the care team. Communicate with the care team. Get familiar with the hospital and the unit. Regularly conduct timed practice sessions with a volunteer.
	**Distractions/obstructions:** Objects, unrelated to the photography, which distract from or obstruct the patient’s skin.	Objects may obstruct part of the skin, visually distract the viewer, and impact the consistency of daily images.	Move items out of frame. Move items off patients’ skin, if able. Drape distracting items.
Communication	**Scheduling:** A missed opportunity to capture uncovered patient’s skin (e.g., dressing change, bath) due to miscommunication between the patient’s care team and photographer or unavailability of the photographer.	Missed dressing changes or baths prevent a complete photograph of the entire skin surface across all body sites from being collected daily.	Communicate daily with the patient’s care team. Ideally, multiple trained photographers should be available. Photographers should have flexible schedules to allow time for sessions when needed.
Patients	Patient wellbeing: The physical or emotional comfort and discomfort of the patient.	Patient wellbeing determines if they are willing to fully participate in repeated photography sessions.	Communicate with the patient and their caretaker. Ask permission to photograph at each session. Explain that the photography session can be stopped at any time. Limit the number of people in the room.
PHI	Protecting PHI & privacy: Photographs may contain sensitive and/or identifying information.	Protecting privacy and PHI helps to establish trust between the patient and photographer.	Cover hospital bands with gauze or tape. Flag photos considered sensitive. Flag photos containing PHI. De-identify photos.
Data management	**Data management:** The organization of photos by establishing standard operating procedures for naming and storing files.	Standardized data management protocol ensures optimal organization, prevents data loss, and makes locating files easier.	Develop a protocol for naming and storing photos. Ensure that filenames are consistent with the naming convention. Keep at least two copies of each photo (have a back-up).
Technical difficulties	**Technical difficulties:** Technological malfunctions due to a loss of power, Wi-Fi, or issues capturing images.	Technical difficulties can prevent data from being collected properly and affect its overall quality.	Use newer-model devices. Fully charge the device before each session. Bring a backup photography device. Confirm all photos are submitted before exiting the photo capture app.

Provided that high-quality photographs are collected, several computer, web-based, and smartphone options for image analysis have been shown to add significant accuracy to BSA assessment (55), enabling completely untrained individuals to outperform experienced providers (56). The application of these technologies could revolutionize the way SJS/TEN studies are conducted by removing time and space constraints in the burn ICU, permitting centralized and standardized quality assurance, and adjudication by off-site experts. A limitation remains the amount of time necessary for a human user to mark borders and otherwise manipulate the photographs in these software interfaces, which can exceed the amount of time to do clinical scoring. One approach is leveraging crowdsourcing of multiple non-expert raters to achieve expert-level accuracy (57), but this would raise issues of patient privacy and data security.

In the future, the application of artificial intelligence (AI) image analysis to standardized photographs could offer practical, rapid, and standardized solutions to the critical gap in SJS/TEN BSA assessments. While there is currently a paucity of literature on this direct application, the SJS/TEN research community can take the following steps to advance:

1. Collating large numbers of standardized SJS/TEN patient photographs, ideally together with clinical variables and patient outcomes
2. Annotating the images with markings of different types of affected skin
3. Connecting these data sets to experts, for example, through global challenges like the melanoma challenge driven by the International Skin Imaging Collaboration (58)

Numerous FDA approvals for medical AI use and even specific guidelines for AI dermatology development (59) and validation lend promise that the combination of photography and AI will eventually lead to substantial advances in SJS/TEN research and patient care. In the near term, higher-quality skin surface assessment and standardized reporting of skin assessment in studies can improve personalized management, prognostic models, and understanding of SJS/TEN. Aside from the limitations stated above, there has been little consensus amongst dermatologists on SJS/TEN terminology, morphological terms and progression and consensus on the most affected sites. A recent study conducted a Delphi consensus exercise to establish a baseline consensus for the development of a standardized SJS/TEN instrument with consistent terminology (60).

## 4. Other considerations for clinical diagnosis and management

### 4.1. SJS mimickers and differential diagnosis

The early features of SJS/TEN are subtle and non-specific with a prodrome of low-grade fever, malaise, anorexia, and mucosal discomfort. It can then progress to include features such as skin pain, and development of bullae, even before the characteristic sloughing of the skin occurs (61). There are many illnesses including infections, autoimmune diseases, and other types of drug reactions that may mimic SJS/TEN (Table 4). Since treatments, prognosis, short and long-term complications, and outcomes vary, prompt and accurate diagnosis is important to guide early intervention and management.

**Table 4:** Most common clinical mimickers of Stevens-Johnson Syndrome & Toxic Epidermal Necrolysis.

Diagnosis	Context	Main clinical difference	Causes
RIME	Abrupt eruption of prominent mucositis triggered by infectious etiologies	Minimal to absent cutaneous eruption, mostly children and young adults	*Mycoplasma pneumoniae* and several other infections
EMM	Development of typical and atypical targetoid macules with central deeper purple or dusky coloration.	Typical, papular 3-zoned targetoid lesions in conjunction with atypical raised targets having only 2 zones, whereas SJS/TEN tends to be flat or flaccid bullous.	Herpes simplex virus most commonly, occasionally other infections, idiopathic, radiation
PNP	Smoldering onset of bullae and lichenoid dermatitis with mucositis, often mistaken for “chronic SJS/TEN”	2 morphologies to eruption: there is both a B-cell mediated bullous morphology and a T-cell mediated lichenoid component	Non-Hodgkin lymphoma, chronic lymphocytic leukemia. Rarely, Castleman’s disease, thymomas, sarcomas, and Waldenström’s macroglobulinemia.
SSSS	Usually newborns, young children, adults with renal failure	Split is very superficial with a periocular, perioral, and intertriginous predilcition. Base of blisters have intact epidermis rather than beefy red dermal appearance. Often intense peri-oral involvement but spares mucous membranes.	Staphylococcal exotoxin (epidermolysin) targeting desmoglein 1
AGEP	Explosive eruption of a brightly erythematous with moist slough	Primary morphology is innumerable, tiny, non-follicularly based pustules on a brightly erythematous base which coalesce to form “lakes of pus.” Time to onset is shorter than SJS/TEN (<4 d), and split is superficial. Absence of mucosal involvement, generally.	Medications
aGVHD4	Morbilliform exanthem that goes on to become blistering, usually within the first 3 months (but can occur later) after transplantation.	Predicliction for dorsal hands and feet, palms and soles, forearms, upper trunk, ears and postauricular areas. GI and hepatic signs/symptoms may be concurrent.	Transplantation of bone marrow, sometimes with multivisceral or small bowel

RIME, Reactive infectious mucocutaneous eruption; EMM, Erythema multiforme major; PNP, Paraneoplastic pemphigus; SSSS, Staphylococcus scalded skin syndrome; AGEP, acute generalized exanthematous pustulosis; aGVHD4, Acute Graft vs. Host Disease, grade IV.

Staphylococcal scalded skin syndrome (SSSS) is a condition with cutaneous involvement that can mimic SJS/TEN. It is a blistering skin condition caused by a toxin from staphylococcus seen either in healthy children with a bacterial focus or in adults with renal insufficiency. SSSS (62) usually presents with tissue-paper thin wrinkling of the epidermis concentrated in intertriginous areas; such as: inguinal folds, axillae, inframammary folds, and folds of the neck. Additionally, peri-oral radial fissures, as well as erythema of the eyes and ears is classic. The skin is red and tender before it sloughs. A very superficial layer of the skin is what sloughs off, revealing a moist, pink, and slightly matte surface at the base, underneath compared to the deep red and shiny exposed dermis that is seen at the base of desquamations in SJS/TEN (61, 62). The skin usually heals completely within 5–7 days after starting treatment with antibiotics and supportive care.

Autoimmune and other immune-mediated disorders comprise an array of diseases that can mimic SJS/TEN. Lupus erythematosus can have many similarities to SJS/TEN. Important differences are photodistribution, and subacute presentation (weeks). Additionally, patients with lupus may have positive antinuclear and reflex-ENA antibodies, elevated anti-dsDNA levels, lymphopenia, and other cytopenia’s and low complement levels which are not typically seen in patients with SJS/TEN (63). Hemophagocytic lymphohistiocytosis (HLH) is a very rare condition caused by natural killer cells and T lymphocytes. It differs from SJS/TEN in that it forms a reticuloform rash and is smoldering, with various stages of resolve although occasionally a positive Nikolsky sign can be seen. Bullous pemphigoid (BP) is a disease that involves the basement membrane. Unlike SJS/TEN, patients with BP will complain of pruritus instead of pain, and their lesions will show a positive Asboe-Hansen sign and a negative Nikolsky sign. Additionally, BP is more often seen in elderly patients without a drug ingestion history. Direct immunofluorescence (DIF) studies of skin reveal linear deposition of IgG and C3 at the basal membrane.

Reactive conditions such as erythema multiforme majus (EMM) are self-limited but occasionally recurrent and may be confused with SJS/TEN. It is hallmarked by typical and/or atypical raised target lesions predominantly on the extremities (acral) in adults and on the face and trunk in children. High fever and several swollen, painful, and erosive mucous membranes may lead to a severe condition in children, whose predominant cause is infection with Mycoplasma pneumonia (64, 65).

Acute graft vs. host disease (GVHD) is a major complication associated with bone marrow transplants. It is a multi-organ disorder that is most commonly due to foreign blood stem cells being transferred to a new host which in turn stimulates an immune reaction. The reaction can be seen following bone marrow transplants, non-irradiated blood transfusions, maternal-fetal transmission, and solid organ transplants. In its most severe form (Stage IV), acute skin disease can consist of generalized involvement with blister formation and skin sloughing resembling SJS/TEN (66).

Several other severe cutaneous adverse drug reactions can present with clinical features mimicking SJS/TEN. These include linear IgA bullous dermatosis, drug-induced hypersensitivity syndrome/drug reaction with eosinophilia and systemic symptoms (DiHS/DRESS) which can present with a wide range of skin morphologies, acute generalized exanthematous pustulosis (AGEP), generalized bullous fixed drug eruption (GBFDE), bullous lichenoid, and multiforme-like drug eruption caused by various medications, and more recently, by the immune checkpoint inhibitors and most commonly PD-1 and PDL-1 inhibitors used in lung cancer. Tumors have evolved to have several mechanisms to cloak themselves from the human immune system. Immune checkpoint inhibitors are used to unharness T and NK cell responses to improve the host tumor response. While this class of medication has been helpful in patient care, it can trigger reactions similar to SJS/TEN.

One last unusual severe cutaneous adverse drug reaction presentation is a delay in the development of a second mucosal site. It has been reported that greater than 85% of patients will present with involvement of two mucosal sites (1, 64). However, we are now becoming aware of a delay in the presentation of the second site in a subset of patients, which may provide initial confusion in the diagnosis.

### 4.2. SJS/TEN and drug-induced liver injury

Significant literature exists that describes the co-existence of drug-induced liver injury (DILI) and SJS/TEN. DILI is the most common cause of acute liver failure in the Western world and is associated with SCARs in 5% of cases. Although DILI most commonly occurs in the setting of DRESS/DIHS, a study looking at 1718 cases of validated DILI, found that 14 patients were diagnosed with concurrent SJS/TEN attributed to 9 different agents (67). The injury pattern in these cases was diverse. Seven presented with hepatocellular injury, while the other seven presented with cholestatic/mixed injury. Most patients presented with a rash and fever but were not jaundiced at the clinical onset but became jaundiced with disease progression. Two patients were classified with mild liver injury, five with moderate injury, and seven with severe injury. Compared with DILI cases, those with concurrent SJS/TEN were more often younger, more likely to be Black, had a shorter latency period from drug exposure to hepatic dysfunction, and ultimately developed a more severe liver injury. While genetic predisposition is suspected, HLA subtyping has not yet demonstrated any clear clinical patterns associated with SJS/TEN co-occurring with DILI. The experience with DILI in the setting of DRESS/DIHS suggests that the same HLA associations may be relevant (68, 69). Physicians diagnosing SJS/TEN should be aware of the possibility of drug-induced liver injury.

### 4.3. Cutaneous toxicities and management of immune checkpoint inhibitor toxicity and SJS/TEN

Immune checkpoint inhibitors (ICIs) such as PD-1, PD-L1, and CTLA-4 inhibitors often lead to non-specific immune activation, of which the skin is the most common target (70–72). Most patients treated with a PD-1 inhibitor will experience at least two or more adverse events (70); fortunately, patients with a cutaneous reaction also demonstrated improved survival rates (73). Common cutaneous adverse events can be classified into psoriasiform, morbilliform, lichenoid eruptions, and vitiligo-like depigmentation (74). Less common adverse events SCARs or blistering dermatoses (74) with the occurrence of an adverse event, the severity of the reaction is categorized utilizing the Common Terminology Criteria for Adverse Events (CTCAE) to communicate the severity of the rash, including total body surface area involved, as well as the safety of reinitiating immunotherapy.

The subtypes of cutaneous adverse events are associated with the type of immune checkpoint inhibitor. Psoriasiform eruptions generally occur with PD1/PD-L1 inhibitors and can be associated with inflammatory joint disease and uveitis. Flares of pre-existing psoriasis are commonly reported, and treatment should resemble a similar therapeutic ladder to classical psoriasis. Morbilliform reactions are the most common adverse event described with CTLA-4 inhibition (75–77). Histopathology typically demonstrates spongiosis, interface dermatitis, and/or perivascular dermatitis with a predominately lymphocytic infiltrate. Treatment is usually limited to the use of topical steroids and oral antihistamines. Lichenoid reactions have an unclear incidence but are more commonly reported with PD-1/PD-L1 inhibitors compared with CTLA-4 inhibitors (78). They are best treated with topical steroids, phototherapy, acitretin, hydroxychloroquine, or apremilast. Vitiligo-like depigmentation does not need therapy, but patients should be educated on the risk of photosensitivity in affected areas. Development of bullous dermatoses is rare, but also likely underreported and underdiagnosed (79, 80). These patients present with a median latency of 6–8 months after PD1/PD-L1 treatment initiation (79, 80). IgG and C3 linear deposits are typically demonstrated on immunofluorescence (80). Considerations for therapy include systemic corticosteroids, dupilumab, omalizumab, intravenous immunoglobulin (IVIG), or rituximab. Lastly, SJS/TEN-like reactions can begin as morbilliform eruptions that evolve into a lichenoid reaction with mucositis of oral, ocular, and genital regions (81, 82). It has recently been suggested that two types of SJS-like eruptions can occur following ICI. Bullous lichenoid reactions, which progress slowly and often occur in the presence of a small molecule drug associated with SCAR, and where rechallenge with ICI may not be contraindicated and reactions appear more like TEN (83, 84). The name progressive immunotherapy-related mucocutaneous eruption (PIRME) has been suggested to refer to these lower acuity reactions which may appear SJS-like but progress more slowly, may have a small molecule culprit drug, and where the pathology suggests a lichenoid bullous reaction (84). Patients then develop full-thickness epidermal necrosis. These patients are best managed in a burn ICU and systemic immunomodulating therapy should be considered.

Although complications of immune checkpoint inhibitor therapy are generally treated with immunosuppression, recent data has demonstrated a significant difference in the overall survival and time to treatment failure with either low or high-dose corticosteroids in patients (85), which sets a precautionary tone. Biomarkers such as IL-6, IgE, and elafin have been correlated with the severity of adverse events, as well as predicted six-month survival (86, 87). A future goal is for a combination of biomarkers and known pathophysiology of the eruption to guide the most judicious and targeted treatment options (87). In addition to corticosteroids, which have been the mainstay of treatment for ICI immune-related adverse events (iRAEs), more targeted therapies, such as etanercept and tocilizumab, are currently being studied and have demonstrated clinical benefit in treating cutaneous immune-related adverse events (88, 89). True severe cutaneous adverse events related to immunotherapy likely have a distinct immunopathogenesis when compared with SJS/TEN related to a small molecule. In addition, ICI may unmask or increase the risk of a SCAR related to a small molecule, such as those described above with lichenoid bullous reactions. Currently, rechallenge is still not recommended with severe cutaneous adverse events related to ICI that mimic and progress rapidly and are similar to SJS/TEN as case reports of fatalities have occurred even with ICI monotherapy rechallenge (90). However, case reports are emerging that may distinguish at least a subgroup of ICI SCAR that appear to tolerate rechallenge with a different ICI (e.g., distinct PD-1 inhibitor) or even the same drug in some instances (84, 91).

### 4.4. Updates on mechanisms

Current innovation in studying gene-protein and T-cell receptor expression at the site of tissue damage in SJS/TEN such as blister fluid and sloughed skin has provided insights into the disease as a CD8-dependent class I HLA-restricted condition with upregulation of markers of cytotoxicity and proliferation. The expression of cytolytic peptides such as granulysin and granzyme B by CD8+ T cells, NK T cells, and NK cells has become the hallmark of SJS/TEN. Examples of how the tissue signatures can be utilized to provide the rationale for successful targeted therapy were exemplified by Kim et al. (92) in the case of a patient with a refractory DiHS/DRESS. Capabilities and the ability to deconvolute and analyze complex datasets are equally important (93, 94).

### 4.5. Cell death pathways and novel therapeutics

SJS/TEN is characterized by the death of keratinocytes. Previously, this epidermal damage in the skin lesions of SJS/TEN patients had been considered to be due to apoptosis. Apoptosis is induced by cytotoxic CD8+ T cells through the Fas–Fas ligand (FasL) pathway or the perforin/granzyme pathway. The cell surface of keratinocytes of TEN patients has revealed a high expression of FasL. In addition, high levels of soluble FasL (sFasL) have been found in the serum of SJS/TEN patients. Fas–FasL interactions mediated apoptosis in the skin lesion of SJS/TEN patients, and in addition, granulysin also demonstrated a cytotoxic effect in SJS/TEN (31). Granulysin, which is found in high levels in SJS/TEN blisters, is released from blister cells in skin lesions of SJS/TEN, including cytotoxic CD8+ T cells, NK T cells, and NK cells. Very recently it has been reported that the exosomal miRNA, miR-375-3p, was markedly upregulated in the plasma of SJS/TEN patients, where it induced mitochondria-dependent apoptosis via downregulation of the X-linked inhibitor of apoptosis protein (XIAP) (95). In 2014, Saito et al. (96) reported that necroptosis induced by annexin A1 – formyl peptide receptor 1 (FPR1) interaction contributes to keratinocyte death in SJS/TEN. In electron microscopic analysis, both necrotic cells and apoptotic cells were observed in the skin lesions of patients. Necroptotic (a type of programmed cell death that reveals morphological necrosis) cells release damage-associated molecular patterns (DAMPs), including a range of pro-inflammatory cytokines, resulting in inflammation, unlike apoptosis (97). The induction of necroptosis in the skin and gut provokes a strong inflammatory response, which might be triggered by the emission of DAMPs (98). In general, necroptosis occurs through the stimulation of TNF-α under conditions in which apoptosis is blocked (97). In TNF-α stimulation, receptor-interacting kinase 1 (RIP1) and receptor-interacting kinase 3 (RIP3) are phosphorylated and form a “necrosome” complex. Furthermore, the mixed lineage kinase domain-like (MLKL) pseudo kinase is recruited to the necrosome and phosphorylated by RIP3. The phosphorylated MLKL (pMLKL) is localized to the plasma membrane and induces cell death (97). Kinoshita et al. (99) discovered neutrophils associated with the mechanism of necroptosis in SJS/TEN. CD8+ T cells produced lipocalin-2, which triggered the formation of neutrophil extracellular traps (NETs) in early lesioned skin. Neutrophils undergoing NETosis released LL-37, and LL-37 induced the expression of FPR1 on keratinocytes through P2X7R stimulation. FPR1 expression caused necroptosis of keratinocytes that caused the further release of LL-37 and induced FPR1 expression on surrounding keratinocytes, which likely amplified the necroptotic response. Necroptosis plays an important role in the immunopathogenesis of SJS/TEN (99). Therefore, inhibition of necroptosis could be an effective therapeutic target. Several compounds, including a new FPR1 antagonist now in development, have been shown to inhibit TEN patient serum-mediated cytotoxicity and keratinocyte death.

Differential gene expression of matrix metalloproteinases (MMPs) and TIMP1 may also predict chronic eye disease in SJS/TEN. In one study, MMP9 was a prognostic predictor of poor best-corrected visual acuity (BCVA) post-cultivated oral mucosal epithelial transplantation (COMET) (100). Another study suggested that epidermal MMP9 expression was significantly higher in SJS/TEN skin than in healthy control skin and non-bullous skin reactions. Serum from SJS/TEN patients also induced MMP9 expression in healthy skin explants which were reduced by etanercept. Furthermore, etanercept reduced TNF-α induced MMP9 expression in cell lines providing additional support for the potential role of etanercept as an SJS/TEN therapeutic agent (101).

Other unexplored areas include the potential for innate triggers for SJS/TEN such as MRGPRX2, a mast cell-specific receptor crucial for pseudo-allergic drug reactions, and the application of novel areas of research such as the field of epigenomics.

Study of particular antigenic epitopes that generate an immune response to specific drugs is of significant interest. This approach has been championed by Kula et al. (102) who described the Tscan^®^ methodology of epitope discovery. Tscan^®^ uses a library screening strategy to validate epitopes of interest. For instance, T cells from an SJS/TEN patient could target cells engineered to carry the human peptidome or virus-specific libraries in addition to the suspected HLA risk allele. Granzyme B-producing cells are sorted and processed by deep sequencing to identify epitopes in conjunction with activated T cells (102).

## 5. Updates in acute care

### 5.1. Updates in supportive care management (Table 5)

**Table 5:** Key points discussed during “updates for clinicians.”

**Specialized units** Consideration should be made to transfer patients with suspected SJS/TEN to hospitals with dermatology inpatient wards or burn centers early in their presentation. The decision should be based on the extent of skin detachment and the need for intensive care. Acute and critical care needs for patients with SJS/TEN can be similar to those of patients suffering a thermal injury. Psychosocial, rehabilitation, and after care needs for patients with SJS/TEN might be better addressed at hospitals with established programs for patients recovering from thermal injury. **Eye care** Early ocular involvement is highly variable and can result in chronic complications leading to severe ocular surface disease including corneal blindness. Patients who receive acute ophthalmic care based on an evidence-based treatment that involves the use of amniotic membrane may be more likely to retain >20/40 vision than those who do not. Customized scleral lenses provide a protective barrier, support the ocular surface, and can prevent corneal complications, improving visual acuity and comfort. **Genitourinary issues** Gynecology was only consulted in half of the cases of possible vulvovaginal involvement. There appeared to be an assumption that there was no need for vulvovaginal care in patients presumably not sexually active. Obtaining consent in a sensitive matter is important in very young/older patients as to explain long-term sequelae. **Unusual presentations** Recognition of SJS/TEN mimickers is critical as management and prognosis can be very different for each category. These include infectious, autoimmune, reactive, and other drug response etiologies. Autoimmune conditions and reactive conditions can produce cutaneous mimics of SJS/TEN but differences exist in presentation, chronicity, laboratory studies and histopathology. While greater than 85% of patients will present with involvement of two mucosal sites some patients have a delayed second mucosal site involvement. Often times this 2nd site includes ocular mucosa.

#### 5.1.1. Burn and critical care management

Acute SJS/TEN is characterized initially by flat, atypical targets or purpuric macules predominantly on the trunk and by mucosal erosions in at least two mucosal sites, often including the ocular surface. Transfer and consultation for patients with SJS/TEN should happen early before advanced critical care is needed. Once progression to multi-organ failure occurs, the transfer of patients may be futile and often leads to a transition to comfort care once they arrive at the tertiary or quaternary hospital with a burn center. These delayed transfers can utilize already scarce resources, distract from the acute management of burn patients, and challenge future collaboration with referring hospitals.

The consensus on how to manage states of shock after burn injury continues to be debated (103). Nonetheless, hospitals with burn programs have extensive expertise in managing non-hemorrhagic hypovolemia. Additionally, some centers have reported that, like burn injury, SJS/TEN may be associated with multifactorial shock. This may include vasodilatory, cardiogenic, and distributive shock phenotypes, and may occur through a perturbed inflammatory stimulation which warrants further investigation. There remains variation by practice on how bullae (or blisters) are managed (104, 105). Some centers remove blisters, while others drain. Most dermatologists prefer to drain bullae that result from SJS/TEN, and therefore collaboration is required between teams to reach a consensus on wound management. Similarly, there is some variability in the selection of topical dressing, which should be a subject of future studies. An international team has just published a Delphi-based consensus paper and wound management was one item examined (11). Regardless of bullae management and dressing choice, wounds should be cleaned and examined for stigmata of infection. If infection concerns arise, topical or/and systemic antimicrobials should be initiated to prevent wound-related infection, and subsequent systemic sepsis. There have been studies examining the effects of grafting the wounds in SJS/TEN after mild wound bed preparation; however, these practices have not become standard in most burn centers (106–108). Re-epithelialization of large areas of skin, either primarily or assisted with grafting, requires significant energy expenditure. Although not studied formally, most burn centers will provide hyperalimentation for patients with SJS/TEN using similar formulae that they would use for patients with burns (109). Burn centers work closely with dieticians and most have them embedded within their teams. Protein calorie malnutrition must be prevented, and assessment of nutritional status should be performed either by indirect calorimetry or adjuncts such as urinary excretion of nitrogen if normal kidney function is maintained. Hypermetabolic states persist after wound closure and need to be monitored similarly to those receiving care for burns. Pharmacotherapies such as propranolol and oxandrolone are currently under study for patients with burns (110, 111), and further work in this area will be needed depending on the results.

### 5.2. Eye care in SJS/TEN

Early ocular involvement is highly variable and not proportionately related to the extent of body surface area detached. It ranges from conjunctival hyperemia to near-total sloughing of the ocular surface, including the tarsal conjunctiva and eyelid margins. Chronic complications can result in severe ocular surface disease including corneal blindness.

For survivors, ocular complications are among the most common and debilitating. In a recent survey conducted at 11 academic health centers in the US which evaluated 121 adults diagnosed with SJS/TEN by inpatient consultive dermatologists, 60% of SJS/TEN patients reported long-term eye problems (112). In another study evaluating 105 eyes of 66 patients, the ocular surface worsened during a follow-up of over 5 years, and more than 50% of eyes with partial conjunctivalization progressed toward total conjunctivalization. The severity of tarsal conjunctival or lid-margin scarring affected the worsening of the ocular surface (113).

All of this points to the critical importance of acute phase management. There is a window of opportunity in the first 7 days to alter visual outcomes. Intervention with the amniotic membrane (AM) is the most critical decision to be made to mitigate eyelid margin disease and prevent the long-term sequelae associated with eyelid microtrauma to the ocular surface (114, 115). Traditionally, AM transplantation (AMT) involved the use of bolsters and sutures to secure AM across the eyelid margin and a symblepharon ring to secure it onto the ocular surface. Recent advances in AMT techniques include using cyanoacrylate glue instead of sutures to secure the AM to the eyelids and allow for a painless and rapid procedure that does not require the use of sedation or general anesthesia. This may be of critical importance in acutely ill patients such as those with SJS/TEN (116).

According to a recent study, patients who receive acute ophthalmic care based on an evidence-based treatment that involves the use of AM were more likely to retain >20/40 vision than those who did not (92% vs.33%). Vision-threatening complications in the chronic phase were also significantly higher in the latter group (67% vs. 17%) (117). However, AMT is not a panacea and long-term complications do still occur, particularly eyelid-related complications and dry eye (118).

Systemic treatments for SJS/TEN have long shown equivocal outcomes in ocular disease. More recently, corticosteroid pulse therapy (CPT), systemic cyclosporine, and etanercept have been explored. In a retrospective case series study by Mieno et al. (119), 36 patients who received CPT within 4 days of disease onset were compared against 49 patients who did not receive such therapy. The percentage of patients with a best corrected visual acuity of 20/200 or greater in the worst eye was significantly different between the two groups, with 52.8% reaching ≥20/200 in those who received CPT vs. 14.3% in those who did not. Severe ocular complications were also significantly less in the group that received CPT. It is important to note that this study was not randomized, so more research may be needed to further validate these findings. Another study evaluated the effects of acute systemic cyclosporine in a small cohort of patients and found no association between the use of systemic cyclosporine therapy and chronic ocular complications (120). Etanercept, however, has been shown, along with concurrent use of AMT, to have a beneficial effect in reducing chronic ocular sequela in a small cohort, though the effects of etanercept vs. AMT may be difficult to separate (121). The question of whether specific acute therapies may be better than others for preventing chronic eye sequelae in SJS/TEN is still an open one.

A pivotal point in the care of chronic ocular disease in SJS/TEN was the introduction of customized scleral lenses known as prosthetic replacement of the ocular surface ecosystem (PROSE^®^). These provide both a protective barrier and support for the ocular surface and can prevent corneal complications, thus improving visual acuity and comfort. PROSE^®^ is often thought of as an intervention that applies only to adults but recently, Wang et al. have shown that pediatric patients with SJS/TEN can also benefit from PROSE^®^ treatment (122). Treatment was feasible in over two-thirds of pediatric patients with chronic ocular surface disease from SJS/TEN and resulted in significant improvements in vision. Other variations of scleral lenses have recently been explored, including a limbal-supported contact lens that led to improved vision compared to spectacles and reduced ocular pain in patients with ocular sequelae from SJS/TEN (123).

Significant advances in our understanding of ocular disease in SJS/TEN have fostered progress in management and outcomes. Though it remains a blinding disease, future advancements will continue to improve vision and visual function in patients with SJS/TEN (124).

### 5.3. Genitourinary disease in SJS/TEN

Although there is consensus on the need that standardized supportive measures should be instituted to prevent long-term genitourinary and reproductive complications in men and women, knowledge of what happens in real clinical practice is lacking. Strictures in the urogenital tract may be more common in women (125). A review of 55 female SJS/TEN survivors sheds light on this issue (126). The key findings from this retrospective review included that gynecology was consulted in <50% of cases and this was unimpacted by the severity of SJS/TEN disease. Furthermore, consultation and care were particularly neglected in girls and young women presumed to be sexually inactive, with no reporting of sexual activity and pregnancy. There was also underutilization of the operating room (OR) and times when sedation was applied to minimize pain and adverse symptoms associated with vulvovaginal exams.

In a subsequent long-term follow-up study involving the same 55 patients, nine patients were found to be deceased, and one patient had an unknown mailing address. Among the remaining 45 patients who were sent follow-up questionnaires, only five patients responded. Although responses were scarce, many noted persistent complaints of vaginal dryness (126).

The overall goal emphasized by this study is the need to standardize the clinical management of women experiencing vulvovaginal sloughing and men with a urogenital disease during the acute phase. It also highlights the importance of improving follow-up care in the gynecology and urology clinics, or alternatively, implementing a multidisciplinary follow-up plan for affected patients.

During the acute phase of SJS/TEN, it is strongly encouraged to consult with gynecology or urology and remain cognizant of potential long-term sequelae such as scarring, strictures, and vaginal dryness. A follow-up plan involving collaboration between different specialties involving gynecologists and urologists is imperative.

### 5.4. Considerations for rehabilitation therapy, hyperproliferative healing, and aftercare reintegration

Physical and occupational therapy is a keystone of burn care and benefits patients with SJS/TEN. Hospitals with burn programs have a higher density of therapists comfortable with managing patients in intensive care units with open wounds. Therapists are also poised to manage anti-deformity positioning and scar prevention. Although not always discussed, patients with SJS/TEN may develop hypertrophic scars that can be remarkably similar to those seen after burn injury (127). Burn therapists are specialists in scar management and employ adjuncts such as splints and compression garments. Acute stress and later post-traumatic stress disorders may develop and burn programs are poised to screen and treat these early. Community, school, and work reintegration are also areas where burn programs have unique expertise and can provide additional resources to patients with SJS/TEN.

### 5.5. Long-term physical and mental health complications of SJS/TEN

Long-term health complications following SJS/TEN are prevalent and underrecognized. SJS survivors have articulated in a recent survey their concerns for inadequacy of post-discharge physical and mental health care (12). Due to incomplete follow-up of SJS/TEN populations, many complications may not have been initially recognized as being associated with SJS/TEN. Recognized complications can include but are not limited to the eye, skin, mucous membrane, ear, internal organ stricture, reproductive, and mental health concerns. One study found that 88.2% of participants felt that their SJS/TEN diagnosis impacted their physical health. In that same study, 70.2% of participants felt that their physicians did not sufficiently address these complications (12).

The acute stage of SJS/TEN is characterized by mucosal membrane involvement (21). Such involvement may include erosion of the ocular mucous membranes. The most feared long-term effects in SJS/TEN are chronic ocular complications. Approximately 50% of SJS survivors report long-term ocular complications (128). Ocular damage can include limbal stem cell deficiency and numerous side effects. Survivors with limbal stem cell deficiency often have epithelial defects, corneal scarring, lid entropion, vascularization, dry eye syndrome, photophobia, corneal abrasions, and erosions due to the corneal epithelium losing the ability to repair itself. Often corneal abrasions and erosions lead to visual impairment, including blindness. According to Gregory (114), “Interventions during the acute stage are crucial, as the long-term sequelae can be difficult, if not impossible, to repair.” Additionally, 77% of SJS/TEN patients present with ocular involvement during the acute stage (129). Standard treatment for SJS/TEN patients can include but is not limited to topical medications, pulse corticosteroid therapy, systemic cyclosporine, symblepharon rings, amniotic membrane transplantation, PROKERA^®^ ring, scleral contact lenses, PROSE^®^ contact lenses, SynergEYES^®^ contact lenses, and limbal supported contact lenses.

It is suggested that daily rinsing of the eyes with sterile saline helps combat inflammatory disease. When used in combination with prophylactic topical antibiotics that are bactericidal, rinsing may also decrease the risk of infection. According to Mieno et al. (119), if given within 4 days of symptom onset, pulse corticosteroid therapy led to significantly better vision and fewer corneal and conjunctival complications. Gregory (114) suggests that systemic cyclosporine may decrease ocular surface inflammation.

Symblepharon may still occur with the treatments above, which indicates the implementation of a symblepharon ring to prevent adhesion of the conjunctiva with the eyelid. In addition, amniotic membrane transplantation may be used for anti-inflammatory and anti-scarring purposes and to promote epithelial healing. Increasing evidence supports a combination of the two previously mentioned treatments, called the PROKERA^®^ ring, which prevents symblepharon, and decreases inflammation and scarring risk while promoting epithelial healing.

Increasing evidence for treatment of chronic eye complications includes, but is not limited to topical medications, scleral contact lenses, PROSE^®^ contact lenses, SynergEYES^®^ contact lenses, and limbal supported contact lenses. SJS/TEN survivors frequently suffer from dry eye syndrome and therefore require constant use of artificial eye drops throughout the day and eye ointment during the night. In addition, some survivors opt to use blood serum tears during the day as they provide healing properties for healthy cell growth and may afford patients additional relief and comfort. Scleral contact lenses are gas-permeable contact lenses designed to cover the eye’s cornea and help with dry eye syndrome. PROSE^®^ contacts provide durable improvements in vision. SynergEYES^®^ contact lenses consist of a stable, rigid center with high oxygen permeability that delivers clear vision and the comfort of a soft lens. Limbal-supported contact lenses are a type of scleral lens that can improve vision and reduce ocular pain. Itoi et al. (123) suggest that wearing limbal-supported lenses improved vision and reduced ocular pain compared to spectacles.

Outside of ocular complications, complications vary in severity as SJS/TEN cases and treatment courses differ among individuals. According to one study, 80% of patients reported skin sequelae from SJS/TEN (128). Skin damage can manifest as hyper-or hypopigmentation, fibrosis, scarring, sealed pores, hair follicle destruction, and nail bed and plate damage. Hyper-or hypopigmentation, fibrosis, and hypertrophic scars are more prevalent in people of color. Survivors with hypertrophic scars may experience sealed pores, leading to overheating in hot weather and the inability to sweat. Additionally, survivors may experience hair follicle destruction causing loss of hair, and many survivors experience damage to their nail beds and plates resulting in slow-growing, fragile, or missing nails.

SJS/TEN can affect the regenerative capacity of the mucosal surfaces. In severe cases, it manifests as scarring/fibrosis. Skin areas exposed to pressure and friction may show delayed healing and sometimes even failure to re-epithelialize. Deeper tissue involvement causes significant damage to progenitor and stem cell populations in affected tissues and can impact the surrounding cellular, immunological, and cytokine microenvironment (130). Hair follicle destruction has also been associated with secondary dermal microcalcifications, scarring, and sebaceous hyperplasia (131).

Many survivors also experience oral health complications, including dental growth abnormalities, low saliva volume (dry mouth), altered tongue, pain, burning sensation, numbness, and loss of taste and smell. Dental growth abnormalities, such as stunted root development, enamel damage, and loss of tooth buds have been observed in children, resulting in missing permanent teeth. SJS/TEN survivors may experience altered tongue, which appears smooth due to filiform and/or fungiform papillae damage. This damage can result in pain, burning sensation, numbness, and loss of taste. Closely related to loss of taste, there may be sinus damage from mucous membrane involvement, resulting in disordered smell perception.

Ear damage can occur which includes scarring and loss of cilia. This can result in complete occlusion of the external auditory canal. Loss of cilia can also lead to abnormal ear wax drainage and loss of hearing.

Urogenital complications most commonly include internal strictures. Female SJS/TEN survivors may experience vulvar, vaginal, and cervical adhesions and scarring, as well as vaginal and cervical stenosis (narrowing) due to damage to mucous membranes which can subsequently complicate childbirth.

Female survivors may also suffer from menstrual disturbances caused by obstruction of the outflow of menstrual blood manifesting as: cyclical abdominal pain, hematocolpos (blood accumulated in the vagina), and hematometra (blood accumulated in the uterine cavity). Both male and female survivors may experience urethral adhesions and scarring, urethral stenosis, hypogastric mass, recurrent painful urination, urinary tract infection, and sexual dysfunction.

Other internal organs can be involved largely from mechanical fractures (strictures) and other organ damage including to the esophagus, colon, liver, renal, gastrointestinal, and respiratory systems. Esophageal strictures commonly manifest as difficulty swallowing. Survivors may also have colon complications such as colitis. Ileal strictures can be associated with chronic diarrhea, intestinal ulcers, intussusception (intestinal inversion), ileal pseudodiverticula, and bleeding. Respiratory complications most commonly include asthma, chronic bronchitis, bronchiolitis obliterations, chronic obstructive pulmonary disease (COPD), interstitial lung disease, pulmonary air leak syndrome, and laryngeal obstruction.

Acute and chronic mental health issues are an important, and often overlooked complication of SJS/TEN that can be prevalent decades later and be a key factor impairing return to work and regular daily activities. Psychiatric damage among survivors can manifest as anxiety and fear of new medicines, survivor guilt, flashbacks, insomnia, depression, and post-traumatic stress disorder. Survivors often feel frustrated due to a lack of providers versed in the disease and a lack of appropriate explanations of how to access specialty care and what to expect. They are particularly fearful of trying new medications and products such as vaccines due to the concern of recurrence.

## 6. Moving the field forward/future directions

SJS/TEN remains a life-threatening and a largely drug-induced disease in adults with high morbidity and mortality. Research into prevention, earlier diagnosis, and treatment of SJS/TEN is impacted by its overall rarity which challenges the ability to study large and diverse populations. The continued development of international networks to synergize efforts from researchers with expertise in different genres of research will be key to the overall success, advancement, and translation. Engagement with the community of SJS/TEN survivors and affected families remains key in this process. Particularly relevant is the fragmentation of healthcare and lack of information on long-term health outcomes for survivors of SJS/TEN. Notable recent advances in SJS/TEN have included insights into earlier diagnosis, mechanisms, risk identification, clinical implications, and pharmaco-surveillance, making risk prediction and prevention possible for some causative factors. As some of the main barriers remain unaddressed, and to truly understand the disease, this research effort requires the collaboration of experts, multidisciplinary leadership/approach, and coordination that includes a critical review of patient-centered clinical and research priorities and unmet evidence-based research needs.

Strengths and opportunities prevail, and in this paper, we have tried to summarize the updated literature on SJS/TEN while highlighting knowledge gaps and research opportunities. Although there have been many recent advances in SJS/TEN research that will improve SJS/TEN outcomes and care, ongoing global research collaboration is urgently needed to address the challenges of studying diverse SJS/TEN populations to include adequate representation of age, gender, race, and ethnicity. Several national and international projects have had small sample sizes that were not ancestrally diverse enough to identify risk alleles, generalized-based risk factors, or effective treatment strategies. These international collaboration networks grown over time will be a powerful vehicle to address unmet needs like developing affordable pharmacogenomic assays, piloting preemptive testing, and incorporating genotypic information that supports the decision-making directly into the medical record which will aid in drug prescription and dispensing systems (Table 6) (6). These networks can also facilitate genome-wide association research studies of other implicated drugs/agents for which robust genomic risk factors are yet to be identified as well as multiomic and mechanistic studies to facilitate the development of earlier diagnostic and prognostic markers and new targeted therapeutic agents.

**Table 6:** Future directions to move SJS/TEN forward.

Unmet needs/gaps	Implementation/focus points
**Prevention, prediction, and regulation:**	
-Lack of knowledge on all casual factors -Generalized genetic test findings -Limited information on casual drugs and targets -Genetic tests with low positive predictive value -Need of evidence-based pre-prescription genetic tests -Lack of real time information on SJS/TEN cases with any new casual drug	-Conduct studies across diverse population groups (age, race, gender, ethnicity) -Low and cost-effective testing -Networks and collaborations to study on multiple drugs, and risk factors -Studies to include genetic and other risk factor identifications -Advancement in pharmacovigilance for immediate updates and alerts on new adverse drug effects
**Early diagnosis and treatment:**	
-Unidentifiable/unreported cases -Inadequate transfer specialized centers -Lack of knowledge on biological markers that aid in early diagnosis -Identify culprit drugs with testing methods (*in vivo/ex vivo/ in vitro*) -Photographic data to assess risk and prognosis	-Clinical awareness and decision-making support -Telehealth triage services -Studies to provide genetic markers and point of care markers for early diagnosis and prognosis -Validate drugs causes across different cohorts -Introduce artificial intelligence algorithms into clinical care
**Clinical care and follow-up:**	
-Need for evidence-based studies to provide best supportive care -Short term treatment plans -Long term clinical/health complications -Coordinated clinical care and support services	-Provide evidence-based study results for best clinical practices -Introduce collaborative networks (domestic and international) in clinical trial studies -Follow-up and long-term care for survivors and families -Coordination among clinical specialties
**Understanding mechanisms and providing care:**	
-Mechanistic studies to identify cellular and molecular signals that act as a biological marker and novel targets for treatment	-Cohort studies on prospectively collected samples for long-term storage with collaborative effort from international networks

## Author’s note

This paper was written using the priority framework of content presented at the virtual meeting: SJS/TEN 2021: Collaboration, Innovation and Community (https://sjsten2021.vfairs.com/).

## Author contributions

MEM and RKB are the co-first-authors of this manuscript. CB, SD, and EJP are the last authors, with EJP as the corresponding author of this manuscript. RKB and MM contributed to the Supplementary Figure. SP, MEM, RKB and EJP contributed to Figure 1. W-HC and MEM contributed to Figure 2. W-CC and CS contributed to Figure 3. ET and MEM contributed to Figure 4. MEM contributed to Figure 5. RKB, MEM, HBP, and KM contributed to Table 1. SH, BK, MM, and RKB contributed to Table 2. MEM and ET contributed to Table 3. HBP contributed to Table 4. JS, TMB, HNS, AS, JC, EF, HBP, and MM contributed to Table 5. RKB contributed to Table 6. All authors listed have made a substantial, direct, and intellectual contribution to the work and approved it for publication.

## Footnotes

^1^https://www.fda.gov/science-research/bioinformatics-tools/fdalabel-full-text-search-drug-product-labeling

## Author disclaimer

TB, MR, and HBP the opinions and assertions expressed herein are those of the authors and do not reflect the official policy or position of the U.S. Food and Drug Administration, Uniformed Services, University of the Health Sciences, or the Department of Defense. This work was prepared by a military or civilian employee of the US Government as part of the individual’s official duties. Therefore, is in the public domain and does not possess copyright protection (public domain information may be freely distributed and copied; however, as a courtesy it is requested that the Uniformed Services University and the author be given an appropriate acknowledgement).
