# Supplementary figures and images for: Updates in SJS/TEN: collaboration, innovation, and community
Source: Front Med (Lausanne). 2023 Oct 11;10:1213889. doi: 10.3389/fmed.2023.1213889 (PMC10600400; doi:10.3389/fmed.2023.1213889)

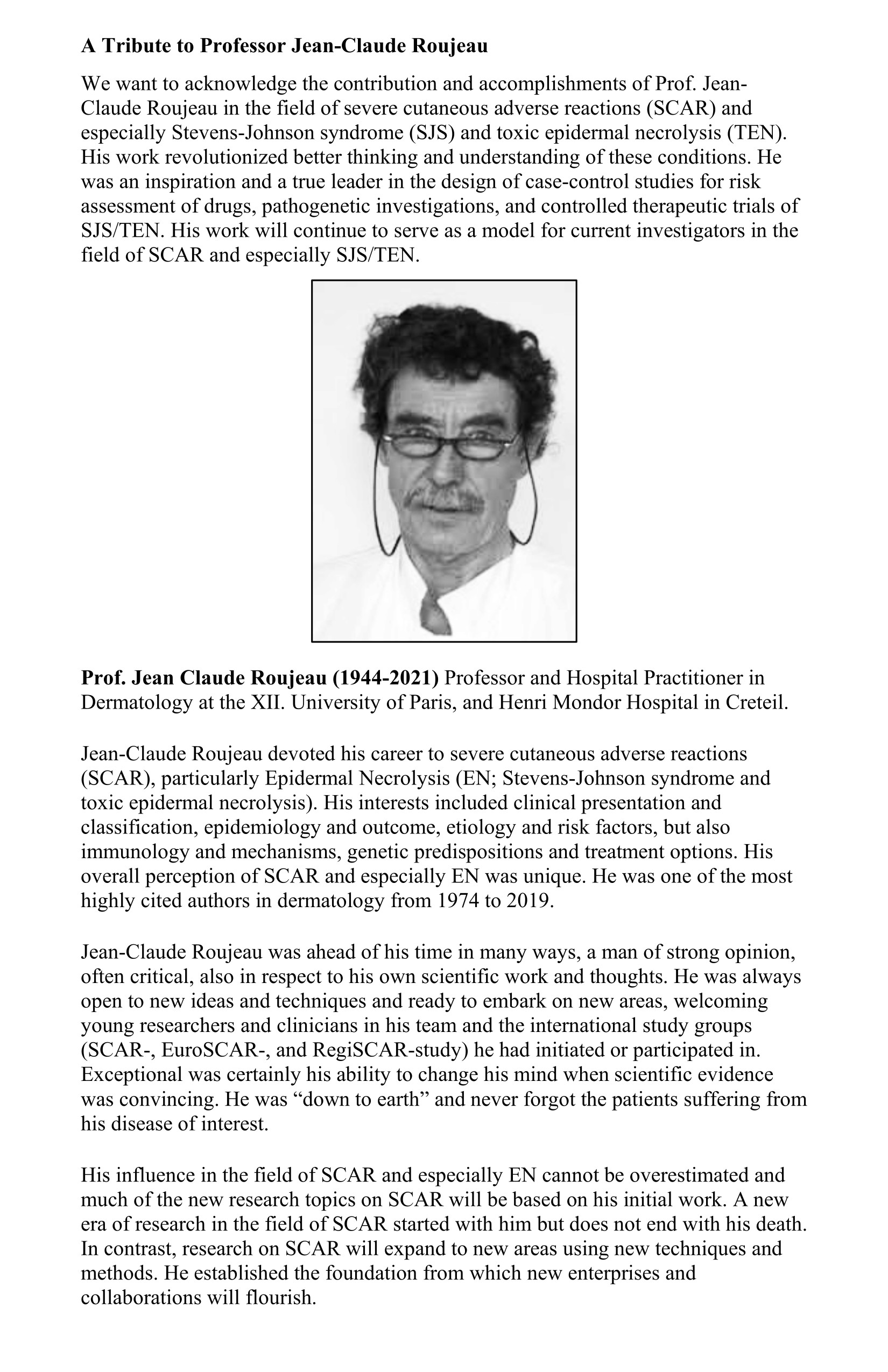

Supplement: Supplementary file 1 [file Image_1.JPEG]
